# Ultrasound-Guided Triamcinolone Acetonide Hydrodissection for Carpal Tunnel Syndrome: A Randomized Controlled Trial

**DOI:** 10.3389/fmed.2021.742724

**Published:** 2021-09-13

**Authors:** Jia-Chi Wang, Po-Cheng Hsu, Kevin A. Wang, Ke-Vin Chang

**Affiliations:** ^1^Department of Physical Medicine and Rehabilitation, Taipei Veterans General Hospital, Taipei, Taiwan; ^2^School of Medicine, National Yang Ming Chiao Tung University, Taipei, Taiwan; ^3^Division of General Surgery, Department of Surgery, Shin-Kong Memorial Hospital, Taipei, Taiwan; ^4^School of Medicine, Fu Jen Catholic University, New Taipei City, Taiwan; ^5^Department of Physical Medicine and Rehabilitation and Community and Geriatric Research Center, National Taiwan University Hospital, Bei-Hu Branch and National Taiwan University College of Medicine, Taipei, Taiwan; ^6^Center for Regional Anesthesia and Pain Medicine, Wang-Fang Hospital, Taipei Medical University, Taipei, Taiwan

**Keywords:** carpal tunnel syndrome, ultrasound-guided hydrodissection, median nerve, injection, triamcinolone acetonide

## Abstract

**Background:** Despite the wide use of corticosteroid hydrodissection for carpal tunnel syndrome (CTS), there is insufficient evidence to confirm its efficacy. This study aimed to compare the effectiveness of corticosteroid hydrodissection vs. corticosteroid perineural injection alone on clinical and electrophysiological parameters in patients with CTS.

**Method:** This prospective randomized controlled trial (RCT) was conducted in a tertiary care center with a follow-up period of 12 weeks. Subjects were randomly assigned to either ultrasound-guided hydrodissection with a mixture of 1 mL of triamcinolone acetonide (10 mg/mL), 1 mL of 2% lidocaine, and 8 mL normal saline or ultrasound-guided perineural injection with 1 mL of triamcinolone acetonide (40 mg/mL) and 1 mL of 2% lidocaine. The primary outcome measure was the symptom severity subscale (SSS) of Boston Carpal Tunnel Questionnaire (BCTQ) scores at baseline and at 6 and 12 weeks' post-treatment. The secondary outcomes included the functional status subscale (FSS) of BCTQ and the distal motor latency and sensory nerve conduction velocity of the median nerve. The effect of interventions on the designated outcome was analyzed using a 3 × 2 repeated measures analysis of variance. The within-subject and among-subject factors were differences in time (before the intervention, and 6 and 12 weeks after injection) and intervention types (with or without hydrodissection), respectively.

**Results:** Sixty-four patients diagnosed with CTS were enrolled. Both groups experienced improvement in the SSS and FSS of BCTQ and median nerve distal motor latency and sensory nerve conduction velocity. However, group-by-time interactions were not significant in any outcome measurements. No serious adverse events were reported in either group, except for two patients in the hydrodissection group who reported minor post-injection pain on the first day after the intervention, which resolved spontaneously without the need for additional treatments.

**Conclusion:** Hydrodissection did not provide an additional benefit compared to corticosteroid perineural injection alone. More prospective studies are needed to investigate the long-term effectiveness of corticosteroid hydrodissection, as well as its influence on median nerve mobility.

## Introduction

Carpal tunnel syndrome (CTS) is the most common entrapment neuropathy of the upper extremity (1). It is caused by compression of the median nerve at the wrist, where it passes beneath the transverse carpal ligament. The most commonly used non-surgical treatments include activity modification, oral medication, wrist splinting, and local corticosteroid injection(s) (2).

The exact pathophysiology of CTS remains elusive (3). Previous studies (4–7) have reported that thickening and fibrosis of the subsynovial connective tissue plays an essential role in the development of CTS and restricts median nerve mobility within the carpal tunnel. A previous systematic review included 10 case-control studies and demonstrated reduced excursion of the median nerve at the proximal wrist among the CTS population compared with normal subjects (8).

Ultrasound-guided hydrodissection is an emerging therapy for entrapment neuropathy described by Malone et al. in 2010 (9). The procedure creates a perineural fluid plane and releases perineural adhesion(s) mechanically. Normal saline, 5% dextrose, and platelet-rich plasma can be used as injectates for hydrodissection (10, 11). By dissecting the subsynovial connective tissue, hydrodissection releases perineural adhesions, which may improve the symptoms of CTS. A cadaver study demonstrated a decrease in gliding resistance of the median nerve in the carpal tunnel after hydrodissection (12).

Local corticosteroid injection has been widely used for the treatment of CTS, with well-established short-term efficacy (13, 14). However, the optimal volume in corticosteroid-containing regimens remain unclear, although it ranges from 1 to 3 mL in most literature reports (14–17). To date, there is no strong evidence supporting whether corticosteroid hydrodissection using a larger volume of injectate(s) provides additional clinical improvement. The purpose of this study, therefore, was to examine whether hydrodissection using corticosteroids could improve pain and function in patients with CTS better than corticosteroid perineural injection alone.

## Method

### Study Design

This was a prospective, single-blinded, randomized control trial in compliance with the Consolidated Standards of Reporting Trials (CONSORT) statement. The study was approved by the Institutional Review Board of Taipei Veteran General Hospital, Taiwan, and was registered at ClinicalTrials.gov (Identifier: NCT04346030). The patients were recruited from the outpatient clinic of the Department of Physical Medicine and Rehabilitation at Taipei Veterans General Hospital. All participants provided written informed consent and were randomly assigned to either intra-carpal corticosteroid injection plus hydrodissection or corticosteroid perineural injection only. Randomization was performed using a computer and random sequence generator in a block of four without stratification. Concealment of treatment assignment was maintained in sealed envelopes that were opened before the interventions.

### Participants

Participants were recruited from outpatient clinics of the Department of Physical Medicine and Rehabilitation at Taipei Veterans General Hospital. The inclusion criteria were as follows: age >18 years; typical symptoms of CTS, including nocturnal-, posture-, or usage-associated paresthesia of the affected hand persisting for at least 3 months; and positive electrophysiological findings with a mild to moderate degree of disease severity (18, 19). The grading system was used in consistent with the serial studies from our laboratory (20, 21). In cases of bilateral involvement, only the side with more severe symptoms was chosen for the intervention. Exclusion criteria were as follows: thenar muscle atrophy or electromyography abnormalities, such as unobtainable median sensory nerve action potential (SNAP) and compound muscle action potential (CMAP), both of which indicate severe CTS (18, 19, 22); coexistence of hypothyroidism, diabetes mellitus, chronic renal failure, rheumatoid arthritis, cervical radiculopathy, polyneuropathy, proximal median nerve entrapment, or thoracic outlet syndrome; previous corticosteroid injection over the affected wrist within the most recent 6 months; previous wrist surgery or distal radius fracture on the side with CTS; pregnancy or lactation; and regular oral intake of non-steroidal anti-inflammatory drugs or corticosteroids.

### Ultrasound-Guided Carpal Tunnel Injection

**Figure 1:**
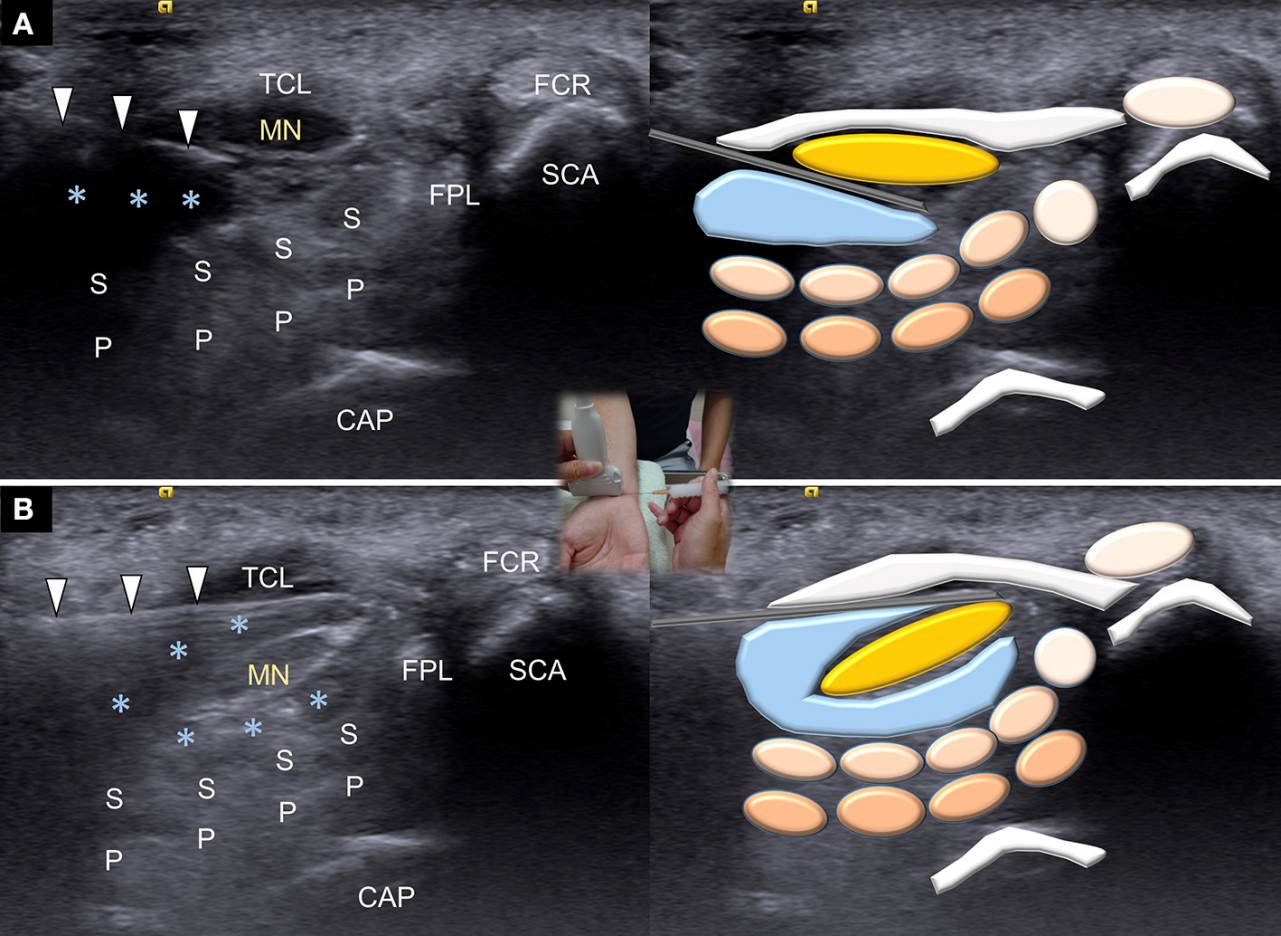
Ultrasound guided hydrodissection for the surface under **(A)** and above **(B)** the median nerve. MN, median nerve; asterisks, injectate; TCR, transverse carpal ligament; FCR, flexor carpi radialis tendon; FPL, flexor pollicis longus tendon; S, flexor digitorum superficialis tendon; P, flexor digitorum profundus tendon; SCA, scaphoid bone; CAP, captitate bone.

All patients received one shot of either ultrasound-guided hydrodissection or corticosteroid perineural injection alone by a single physiatrist, using an Acuson S2000 device (Siemens Healthcare, Erlangen, Germany) equipped with a linear 9–12 MHz probe. The board-certificated physiatrist who performed the intervention had more than 10 years in practicing diagnostic and interventional ultrasound for musculoskeletal medicine. The ultrasound guidance had been applied on the procedures in both groups. Patients were in a sitting position with the hand resting on a table, and the injected wrist was supinated and slightly dorsiflexed. The transducer was placed transversely at the proximal carpal inlet. After sterile preparation, a 23-gauge, 1.5-inch needle was inserted using the in-plane approach superficial and radial to the ulnar artery and nerve. The injection [group 1, 1 mL 2% lidocaine hydrochloride + 1 mL triamcinolone acetonide (10 mg/mL) + 8 mL normal saline; group 2, 1 mL 2% lidocaine hydrochloride + 1 mL triamcinolone acetonide (10 mg/mL)] was administered to the affected median nerve from its ulnar aspect. Lidocaine hydrochloride was added in the injected regimens for both groups for decreasing post-injection discomfort. In the group receiving corticosteroid perineural injection, the injectate was introduced when the needle tip was advanced besides the median nerve (23). In the hydrodissection groups, one-half of the injectate was delivered to the area above the nerve and one-half to the area below the nerve (12) (Figure 1). During hydrodissection, separation of the subsynovial tissues had been visually confirmed between either the median nerve and flexor retinaculum or between the median nerve and underlying finger flexor tendons. The injectates were seen encircling the nerve like a halo with more injected fluid accumulated at the nerve's ulnar side. The procedure time was around 3 min for the hydrodissection technique and within 1 min for perineural corticosteroid injection only.

### Outcome Measurement

The primary outcome was based on the symptom severity score (SSS) of Boston Carpal Tunnel Questionnaire (BCTQ) scores (24). The patients were evaluated at baseline and re-assessed at 6 and 12 weeks after the intervention. The secondary outcomes included the functional severity score (FSS) of BCTQ and the median nerve distal motor latency (DML) and median sensory nerve conduction velocity (SNCV) from the electrophysiological study, which served as additional data to help interpret the results of the primary outcome. We did not treat the secondary outcome alone to interpret the comparative effectiveness between both interventions. The data reporting and interpretation were in accordance to a recent guideline published in 2019 (25). All assessments were performed by another assessor who was blinded to treatment allocation.

#### BCTQ

The BCTQ is a self-administered questionnaire that was used to assess the severity of symptoms and functional status (26). It consists of 19 items divided into two distinct scales: SSS (11 items) and FSS (8 items). Each item was scored from 1 (no complaints) to 5 (very severe complaints) points. The overall SSS and FSS scores were calculated using the mean of the responses to the individual items.

#### Electrophysiological Studies

Electrophysiological studies were performed at baseline and 12 weeks after injection in accordance with the American Association of Electrodiagnostic Medicine guidelines using an electromyography measuring system (Neuropack MEB-9200J, Nihon Kohden, Shinjuku City, Tokyo, Japan). CMAP and DML were obtained using surface electrodes placed on the abductor pollicis brevis muscle, and the median nerve was stimulated 8 cm proximal to the active recording electrode. SNAP and SNCV were obtained using surface electrodes placed at the proximal and distal interphalangeal joints of the index finger. The sensory nerve was stimulated at the wrist at a distance of 14 cm from the wrist to the active electrode. Median sensory latency was measured as the peak.

### Statistical Analysis

The sample size was estimated in an antecedent pilot study investigating betamethasone injection for CTS (27). A standardized mean difference of 0.3 was assumed between different therapeutic arms, with a standard deviation of 0.4, an alpha level (α) of 0.05, and a power (β) of 80%. A total of 58 participants should be at least enrolled.

Data normality was assessed using the Shapiro-Wilk test. Univariate analysis of continuous variables was performed using one-way analysis of variance or the Mann-Whitney *U*-test (if the data were not normally distributed). The chi-squared or Fisher's exact tests (in the case of sparse data) were used to compare categorical data. The effect of interventions on the designated outcome was analyzed using a 3 × 2 repeated measures analysis of variance. The within-subject and among-subject factors were differences in time (before the intervention, and 6 and 12 weeks after injection) and intervention types (with or without hydrodissection), respectively. The analyses were conducted by employing MedCalc 14.0 (MedCalc Software, Ostend, Belgium) statistical software; *p*-values < 0.05 were considered statistically significant.

## Results

### Participants

**Figure 2:**
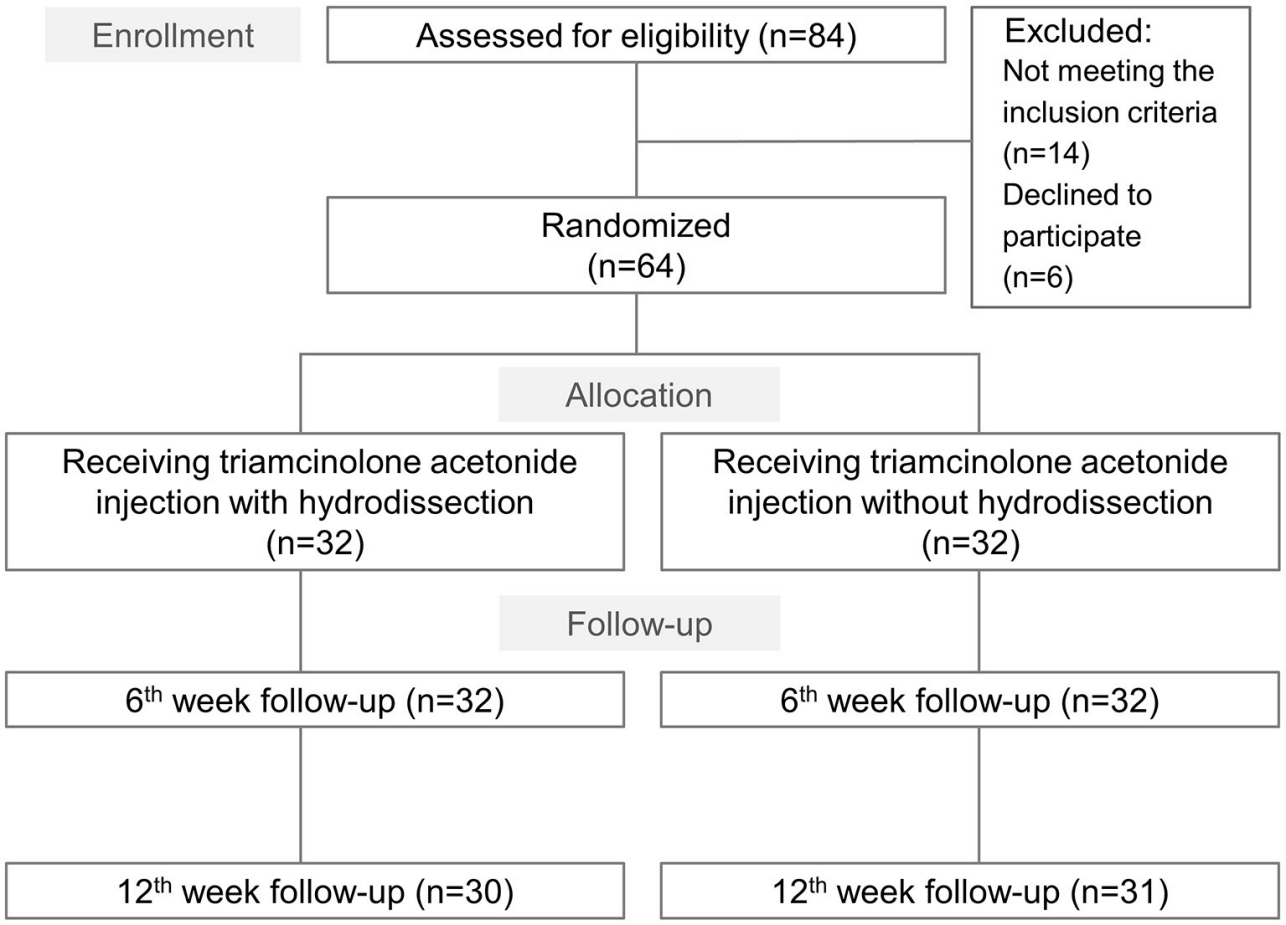
Study flow diagram for the group receiving ultrasound guided triamcinolone acetonide injection with hydrodissection and that undergoing triamcinolone acetonide injection alone.

A total of 84 individuals potentially eligible for assessment were invited to participate in the trial, of whom 64 ultimately provided informed consent and randomly assigned to a group receiving hydrodissection (*n* = 32) and another without hydrodissection (*n* = 32) (Figure 2). Of these, three patients were lost to follow-up at 12 weeks' post-injection. No significant between-group differences were observed in the proportion of participants lost to follow-up. Baseline characteristics and outcome variables, including age, sex ratio, body mass index, hand dominance, symptom duration, smoking status, BCTQ, and electrophysiological findings were not significantly different between the groups (Table 1).

**Table 1:** Baseline characteristics of the patients.

	**Group 1** **(** ***n*** **= 32)**	**Group 2** **(** ***n*** **= 32)**	***p*** **-value**
Age (years)	52.87 ± 10.19 (49.19–56.55)	53.28 ± 9.67 (49.79–56.76)	0.871
Gender (F/M)	24/8	28/4	0.206
Dominant side (R/L)	32/0	32/0	
Duration of symptoms (month)	22.93 ± 25.87 (13.60–32.26)	24.31 ± 31.85 (12.82–35.79)	0.850
Smoking (number)	0	2	0.156
Boston Carpal Tunnel Questionnaire			
SSS	2.13 ± 0.60 (1.91–2.34)	2.16 ± 0.61 (1.93–2.38)	0.839
FSS	1.76 ± 0.54 (1.56–1.96)	1.81 ± 0.72 (1.55–2.07)	0.787
Nerve conduction study			
DML (ms)	5.58 ± 1.59 (5.01–6.16)	5.38 ± 1.32 (4.90–5.85)	0.524
SNCV (m/s)	36.26 ± 11.51 (32.11–40.41)	38.83 ± 9.59 (35.38–42.29)	0.335

*Data are given as mean ± standard deviation (95% confidence interval of mean)*.

*Group 1, hydrodissection with corticosteroid; Group 2, corticosteroid injection alone*.

*F, female; M, male; R, right; L, left; SSS, symptom severity scale; FSS, functional status scale; DML, distal motor latency; SNCV, sensory nerve conduction velocity*.

### Outcome Measurements

**Figure 3:**
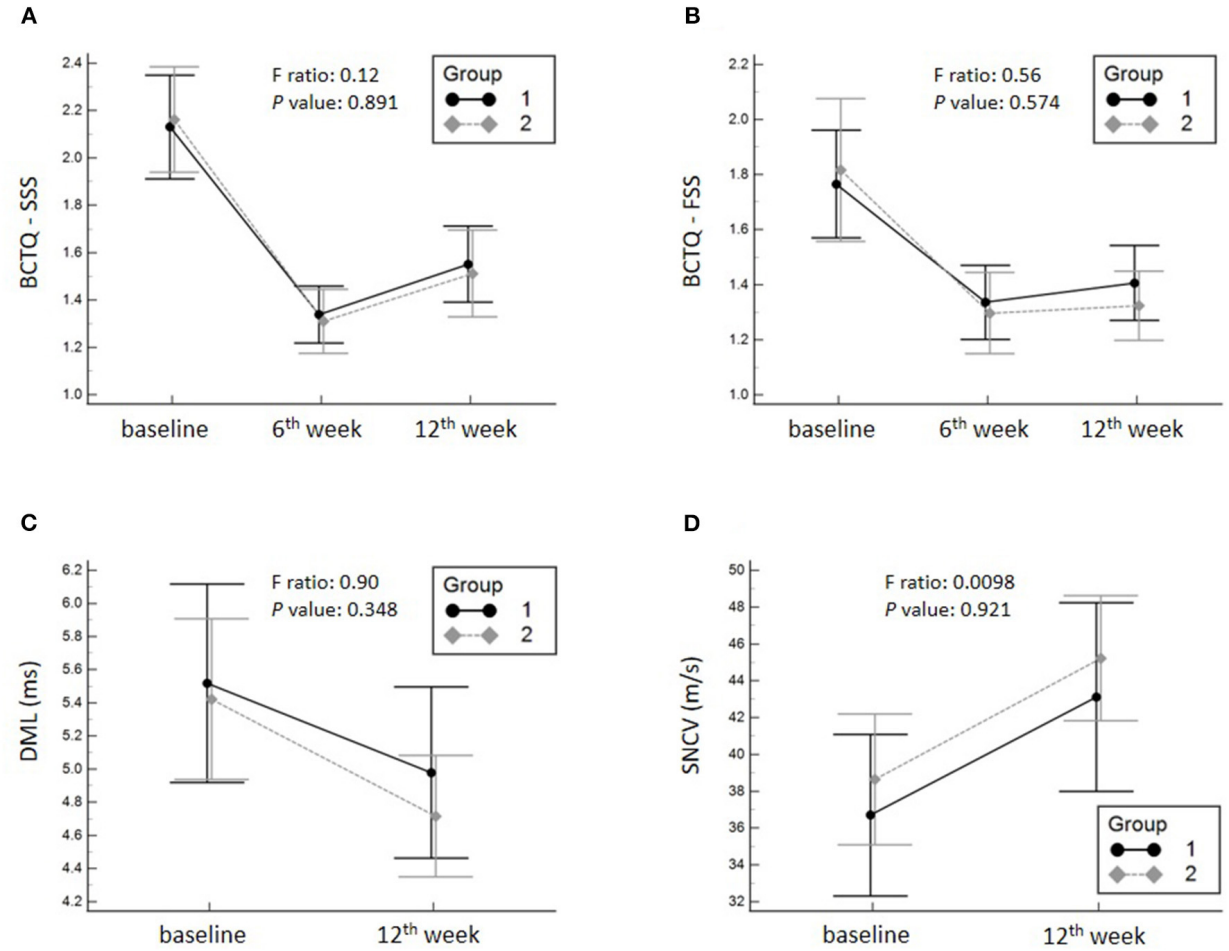
Mean changes and the corresponding 95% confidence intervals pertaining to the symptom severity scale (SSS) **(A)** of the Boston Carpal Tunnel Questionnaire (BCTQ), functional status scale (FSS) **(B)** of the BCTQ and distal motor latency (DML) **(C)** and sensory nerve conduction velocity (SNCV) of the median nerve **(D)**. Group 1, hydrodissection with corticosteroid; Group 2, corticosteroid perineural injection alone.

A significant decrease in the SSS and FSS subscales of the BCTQ and DML, and an improvement in SNCV were observed in both groups at 6 and 12 weeks after intervention compared with the baseline condition. Regarding the primary outcome, no significant group-time interaction was identified in terms of the SSS (F ratio: 0.12; *p*-value: 0.891) of BCTQ. Likewise, there was no significant group-time interaction of the FSS of BCTQ, DML and SNCV of the median nerve. The details of between-group comparisons are summarized in Table 2; Figure 3.

**Table 2:** Clinical Outcomes at baseline, 6th and 12th week.

	**Group 1** **(** ***n*** **= 32)**	**Difference from baseline**	**Group 2** **(** ***n*** **= 32)**	**Difference from baseline**	***p*** **-value**	**Cohen's** ***d***
**Boston Carpal Tunnel Questionnaire – symptom severity scale**						
Baseline	2.13 ± 0.60^a^^b^ (1.91–2.34)	–	2.16 ± 0.61^a^^b^ (1.93–2.38)	–	–	–
6th week	1.33 ± 0.33^ac^ (1.21–1.45)	−0.79 ± 0.62 (−1.01 to −0.56)	1.30 ± 0.37^a^ (1.17–1.44)	−0.85 ± 0.69 (−1.10 to −0.60)	0.720	0.216
12th week	1.55 ± 0.44^b^^c^ (1.39–1.71)	−0.57 ± 0.63 (−0.80 to −0.35)	1.51 ± 0.50^b^ (1.32–1.69)	−0.65 ± 0.74 (−0.91 to −0.38)	0.682	0.210
**Boston Carpal Tunnel Questionnaire – functional status scale**						
Baseline	1.76 ± 0.54^a^^b^ (1.56–1.96)	–	1.81 ± 0.72^a^^b^ (1.55–2.07)	–	–	–
6th week	1.33 ± 0.37^a^ (1.20–1.47)	−0.42 ± 0.53 (−0.62 to −0.23)	1.29 ± 0.41^a^ (1.14 to 1.44)	−0.51 ± 0.59 (−0.73 to −0.30)	0.547	0.172
12th week	1.40 ± 0.37^b^ (1.27–1.54)	−0.35 ± 0.52 (−0.54 to −0.17)	1.32 ± 0.34^b^ (1.19–1.45)	−0.49 ± 0.59 (−0.70 to −0.27)	0.716	0.136
**Distal motor latency** **(m/s)**						
Baseline	5.58 ± 1.59^b^ (5.01–6.16)	–	5.38 ± 1.32^b^ (4.90–5.85)	–	–	–
12th week	4.97 ± 1.38^b^ (4.46–5.49)	−0.53 ± 0.70 (−0.80 to −0.27)	4.71 ± 0.99^b^ (4.35–5.08)	−0.70 ± 0.67 (−0.95 to −0.45)	0.228	0.225
**Sensory nerve conduction velocity** **(m/s)**						
Baseline	36.26 ± 11.51^b^ (32.11–40.41)	–	38.83 ± 9.59^b^ (35.38–42.29)	–	–	–
12th week	43.11 ± 13.70^b^ (37.99–48.23)	6.41 ± 8.62 (3.19–9.63)	45.22 ± 9.24^b^ (41.83–48.61)	6.59 ± 4.96 (4.76–8.41)	0.175	0.232

*Data are given as mean ± standard deviation (95% confidence interval of mean). P values pertain to between-group comparisons for differences from baseline*.

*Group 1, hydrodissection with corticosteroid; Group 2, corticosteroid injection alone*.

a *Significant difference between baseline and the 6th week in the same group*.

b *Significant difference between baseline and the 12th week in the same group*.

c *Significant difference between the 6th and 12th week in the same group*.

### Adverse Events

Every patient tolerated either procedures well and no participant asked for stopping the treatment in the middle of the intervention. No serious adverse events were reported in either group, except for two patients in the hydrodissection group who reported minor post-injection pain on the first day after the intervention, which resolved spontaneously without the need for additional treatments.

## Discussion

The present clinical trial yielded several important observations. First, corticosteroid injection with or without additional hydrodissection led to a significant decrease in pain, symptoms, functional impairment, and electrophysiological abnormalities in patients with CTS. Second, there was no difference in clinical effectiveness between corticosteroid injection with and without hydrodissection, based on evidence from our RCT.

Various injectates have been used for nerve hydrodissection, including normal saline, corticosteroid, 5% dextrose in water (D5W), and platelet-rich plasma (10). One published RCT assessed the effect of different volumes of D5W on the treatment of CTS (28). Lin et al. compared 1, 2, and 4 mL of D5W injection in patients with CTS and found that 4 mL of D5W was more effective than 1 and 2 mL in symptom and functional improvement at 1, 4, and 12 weeks' post-injection (28). However, the superiority of the clinical effectiveness of 4 mL of injectate declined at 24 weeks post-injection.

Corticosteroids are the most commonly used injectates for CTS. Its mechanism of action is mainly through anti-inflammatory reactions, with a decrease in the number of lymphocytes, as well as inhibition of cytokine expression and phospholipase activation (29). Furthermore, corticosteroids also reduce vasogenic edema by affecting the permeability of the vascular wall (30). Previous studies evaluating the efficacy of corticosteroid hydrodissection have been limited to case series without a control group or a comparative therapeutic arm (9, 31). Malone et al. treated 44 wrists in 34 patients with CTS using ultrasound-guided hydrodissection of the median nerve [9 mL normal saline, 1 mL 1% lidocaine, and 1 mL triamcinolone acetonide (40 mg/mL)] and fenestration of the flexor retinaculum, yielding complete symptom resolution in 30 wrists at an average of 8 months post-intervention (9). DeLea et al. managed 14 patients with CTS related to rheumatoid arthritis and 12 with painful scleroderma of the hand using ultrasound-guided hydrodissection (3 mL 1% lidocaine and 80 mg triamcinolone acetonide). At the second post-injection week, they found a 47 and 67% reduction in pain scores in the former and latter groups, respectively (31).

Through our literature search from PubMed and EMBASE, we could only find one other paper with similar methodology. The small RCT was designed to investigate the influence of corticosteroid hydrodissection on CTS (27). Schrier et al. enrolled 20 patients randomly assigned to either ultrasound-guided hydrodissection using 1 mL of betamethasone (6 mg), 1 ml of 1% lidocaine, and 3 mL saline or injection only with 1 mL of betamethasone (6 mg) and 1 mL of 1% lidocaine (27). The results revealed no significant differences in the visual analog scale of pain or BCTQ scores between the groups at 24 weeks after the intervention. Therefore, our RCT was complementary to the aforementioned study, whose clinical evidence was limited by the small number of cases. The comparisons of the demographic and intervention characteristics between ours and the aforementioned study are summarized in Tables 3, 4, respectively.

**Table 3:** Comparison of the demographic characteristics between the current study and another randomized controlled trial with similar methodology.

**References**	**Study design**	**Inclusion criteria**	**Treatment allocation**	**Participant characteristics**			**Symptom duration (months)**	**Disease severity**	**Randomization**	**Blinding**	**Outcome measure**	**Follow-up (week)**
				**Number of participants**	**Mean age (year)**	**Female (%)**						
Wang et al. (the current study)	RCT	Clinical + EDS	Corticosteroid hydrodissection	32	52.87 ± 10.19	75%	22.93 ± 25.87	Mild to moderate	Computer-generated randomization	Blinded outcome assessors	BCTQ, VAS (not reported), EDS	6, 12
			Corticosteroid Injection alone	32	53.28 ± 9.67	87.5 %	24.31 ± 31.85					
Schrier et al. (27)	RCT	Clinical + EDS	Corticosteroid hydrodissection	11	47^#^ (37–61)	66.6 %	12^#^ (6.5–14)	Mild to moderate	Computer-generated randomization	Blinded outcome assessors	BCTQ, VAS, EDS, CSA of MN, displacement of MN	4, 24
			Corticosteroid Injection alone	8	59^#^ (55–66)	90.9%	12^#^ (7–96)					

*BCTQ, boston carpal tunnel syndrome questionnaire; VAS, visual analog scale; PRP, platelet-rich plasma; D5W, 5% dextrose; NA, not available; RCT, randomized controlled trial; EDS, electro-diagnostic study; CSA, cross-sectional area; MN, median nerve*.

*The data are presented as n or mean ± standard deviation*.

# *Indicates that the data are presented as median (25–75th percentiles)*.

**Table 4:** Comparison of the intervention characteristics between the current study and another randomized controlled trial with similar methodology.

**References**	**Intervention**	**Intervention regiment**	**Guidance method**	**Injection site**	**Adverse effect**
Wang et al. (the current study)	Hydrodissection vs. Perineural injection	1 mL of 2% lidocaine + 1 mL triamcinolone (10 mg) + 8 mL saline vs. 1 mL of 2% lidocaine + 1 mL triamcinolone (10 mg)	Ultrasound–guided ulnar in-plane approach	Proximal carpal tunnel inlet.	No complication
Schrier et al. (27)	Hydrodissection vs. Perineural injection	1 mL of betamethasone (6 mg), 1 mL of 1% lidocaine and 3 mL saline vs. 1 mL of betamethasone (6 mg) and 1 mL of 1% lidocaine	Ultrasound-guided ulnar in-plane approach	Underneath the transverse carpal ligament	No complication

Based on evidence from our RCT, we did not observe any significant difference in clinical effectiveness between the two groups after the intervention. We believe that a total of 10 mL was sufficient for the hydrodissection of perineural adhesions. Complete separation of the median nerve from the surrounding tissues was confirmed along the entire nerve length in the carpal tunnel in our study. Furthermore, an increase in intra-carpal tunnel pressure is a pathogenic factor of CTS (3). The volume (10 mL) used in our study is known to be the highest among the existing literature (10). It is possible that such a high volume within the carpal tunnel may elevate intra-carpal tunnel pressure and counteract the benefit(s) of hydrodissection.

Regarding the dose of corticosteroid, most reports in the literature describe injections of 20–40 mg triamcinolone acetonide or an equivalent potency dosage of methylprednisolone for treating CTS (32–35). The dose of triamcinolone acetonide (10 mg) used in the present study was small, which facilitated the comparison of the add-on benefit of hydrodissection. A study by Peters-Veluthamaningal et al. reported that one or two intra-carpal tunnel injections with 10 mg triamcinolone was more effective than placebo injections for CTS (36) in a short blinded follow-up period. A recent RCT confirmed that a single 10 mg triamcinolone acetonide injection was as effective as 40 mg for CTS management (21). In addition, both groups in the present study received the same dosage of corticosteroid, which theoretically had minimal impact on the between-group comparison of clinical effectiveness. Furthermore, because our chosen corticosteroid type was triamcinolone, potential toxicity of peripheral nerves (37) should be considered in case of accidental intra-neural injection. The aforementioned reasons made us choose 10 mg triamcinolone at the first place. However, an antecent RCT (38) pointed out that 80 mg methylprednisolone was better than 40 mg methylprednisolone in improving symptoms of CTS and decreasing the rate of needing for surgeries. Therefore, a higher dose of triamcinolone, like 40 mg, should be prioritized as the treatment regimen for hydrodissection, although extra caution should be taken during injections for prevention of neurotoxicity.

## Limitations

Our study had some limitations, the first of which was the short follow-up period of only 12 weeks. Based on the results of most literature, corticosteroid injections for CTS only provide short-term benefits. In a retrospective analysis of 233 patients treated with intracarpal corticosteroid injections, Green et al. (39) reported that the majority of the patients displayed recurrent symptoms at 2–4 months after the initial treatment. In a prospective study, Gelberman et al. (40) reported that the maximal improvement of CTS symptoms occurred at 1–2 months after corticosteroid injections. Armstrong et al. (41) reported that after a median interval of 103 days following the initial corticosteroid injection, more than 50% of patients requested a second injection for recurrent symptoms. In a systemic review, Marshall et al. (14) concluded that the effect of corticosteroid injection beyond 1 month remained uncertain. Moreover, our previous study (42) revealed the effects of intracarpal corticosteroid (10 mg triamcinolone) declined at the post-injection 6 weeks. Therefore, we chose 12 weeks to evaluate if hydrodissection had an add-on benefit to corticosteroid injections for managing CTS. However, further prospective studies with longer follow-up periods to obtain the rates of relapsing and needing for surgeries are still needed.

Second, the study did not include a placebo group; thus, the effect of hydodissection or corticosteroid perineural injection using a small volume compared with the natural course could not be determined through our analysis. Third, the cross-sectional area, flattening ratio and stiffness of the median nerve have been identified to be different between patients with CTS and those without by ultrasound imaging. A previous meta-analysis (43) has demonstrated that the diagnostic odd ratio of the nerve cross-sectional area could reach 31.11 [95% confidence interval (CI), 20.42–47.40] over the carpal tunnel inlet, whereas another meta-analysis (44) has shown that the sensitivity and specificity for diagnosing CTS could be up to 0.94 (95% CI, 0.90–0.97) and 0.80 (95% CI, 0.71–0.88), respectively, by evaluating nerve stiffness using shear wave sonoelastography. Regarding the adhesion of subsynovial connective tissue, Park et al. (45) revealed a reduction of median nerve gliding employing dynamic ultrasound imaging in the CTS group. Through reviewing the aforementioned meta-analyses (43, 44) and original study (45), the use of ultrasound image has gradually become an alternative modality for the diagnosis and follow-up of CTS. Furthermore, clinical assessments, such as the grip and pinch strength (46), performance of fine motor control (47), and two-point discrimination (48) would be helpful in providing objective data. Nevertheless, we did not incorporate the aforementioned ultrasound and clinical assessments at the beginning of the study, which should be encompassed in future relevant research. Fourth, the Likert scale (49), a psychometric measurement, is widely used for assessing the participant's agreement with various statements. However, we did not include this scale to evaluate their acceptability/satisfaction for both interventional modalities in the beginning of this RCT, which should be added in our subsequent pertinent study. Fifth, the addition of the local anesthetics in the injectates could possibly interfere the estimation of the treatment effect and also elicited temporary numbness of the region innervated by the median nerve distal to the carpal tunnel. Therefore, there is no need to add local anesthetics in future clinical practice. Sixth, the grading system for CTS severity (18) used in this research might be out-of-date and a modern severity grading tool (50, 51) should be adopted in our next trial.

## Conclusion

Ultrasound-guided hydrodissection using triamcinolone acetonide and corticosteroid perineural injection alone both resulted in clinical and electrophysiological improvement in patients with CTS at 12 weeks' post-treatment. However, additional hydrodissection did not provide additional benefit compared with corticosteroid injection perineural alone. More prospective studies are needed to investigate the long-term effectiveness of corticosteroid hydrodissection, as well as its influence on median nerve mobility.

## Data Availability Statement

The raw data supporting the conclusions of this article will be made available by the authors, without undue reservation.

## Ethics Statement

The studies involving human participants were reviewed and approved by Institutional Review Board of Taipei Veteran General Hospital. The patients/participants provided their written informed consent to participate in this study.

## Author Contributions

J-CW and K-VC: conceptualization, methodology, resources, funding acquisition, and formal analysis. P-CH: software. K-VC: investigation, supervision, and writing—review and editing. P-CH and KW: data curation and visualization. J-CW and KW: writing—original draft preparation. J-CW and P-CH: project administration. All authors contributed to the article and approved the submitted version.

## Funding

This study was made possible by the research funding of the Community and Geriatric Medicine Research Center, National Taiwan University Hospital, Bei-Hu Branch, Taipei, Taiwan; Ministry of Science and Technology (MOST 106-2314-B-002-180-MY3, 109-2314-B-002-114-MY3, and 109-2314-B-002-127), and Taiwan Society of Ultrasound in Medicine.

## Conflict of Interest

The authors declare that the research was conducted in the absence of any commercial or financial relationships that could be construed as a potential conflict of interest.

## Publisher's Note

All claims expressed in this article are solely those of the authors and do not necessarily represent those of their affiliated organizations, or those of the publisher, the editors and the reviewers. Any product that may be evaluated in this article, or claim that may be made by its manufacturer, is not guaranteed or endorsed by the publisher.

## Abbreviations

CTS: Carpal tunnel syndrome
BCTQ: Boston Carpal Tunnel Questionnaire
SSS: Symptom severity scale
FSS: Functional severity scale
VAS: Visual analog scale
SNAP: Sensory nerve action potential
CMAP: Compound muscle action potential
DML: Distal motor latency
SNCV: sensory nerve conduction velocity.
